# Dispersion-Controlled Carbon Nanotube Networks Enable Tunable Structure–Performance–Stability Relationships in RuO_*x*_·*n*H_2_O Pseudocapacitive Electrodes

**DOI:** 10.1021/acsomega.6c05011

**Published:** 2026-09-14

**Authors:** Wilson Hou-Sheng Huang, Yuli Lin, Wen-Jin Li

**Affiliations:** † Center for Continuing Education, 34881 National Tsing Hua University, No.101, Section 2, Kuang-Fu Rd., Hsinchu City 300044, Taiwan R.O.C; ‡ Department of Optoelectronics and Material Engineering, 63119 Chung Hua University, 707, Sec. 2, WuFu Rd., Hsinchu City 30012, Taiwan R.O.C; § Material and Chemical Research Laboratories, 63129 Industrial Technology Research Institute, No. 195, Sec. 4, Zhongxing Rd., Zhudong Township, Hsinchu County 310401, Taiwan R.O.C

## Abstract

The electrochemical performance of pseudocapacitive electrodes is closely related to their nanoscale structure and conductive network formation. In this study, hydrous RuO*_x_* ·nH_2_O electrodes with controlled carbon nanotube (CNT) dispersion were fabricated via a cathodic deposition method to investigate structure–performance relationships. Dispersed CNT form interconnected conductive networks that improve electron transport and active material utilization, resulting in a high specific capacitance of 718 F g^–1^. Long-term cycling over 100,000 cycles reveals a clear trade-off between performance and stability, with nearly 100% capacitance retention for CNT-free electrodes and approximately 40% for dispersed CNT systems. This behavior is associated with the formation of more distributed RuO*_x_* ·nH_2_O coatings on CNT frameworks, which influence structural stability during repeated cycling. These results demonstrate that CNT dispersion plays an important role in determining electrode structure and electrochemical performance, providing useful insights for the design of pseudocapacitive materials.

## Graphical abstract

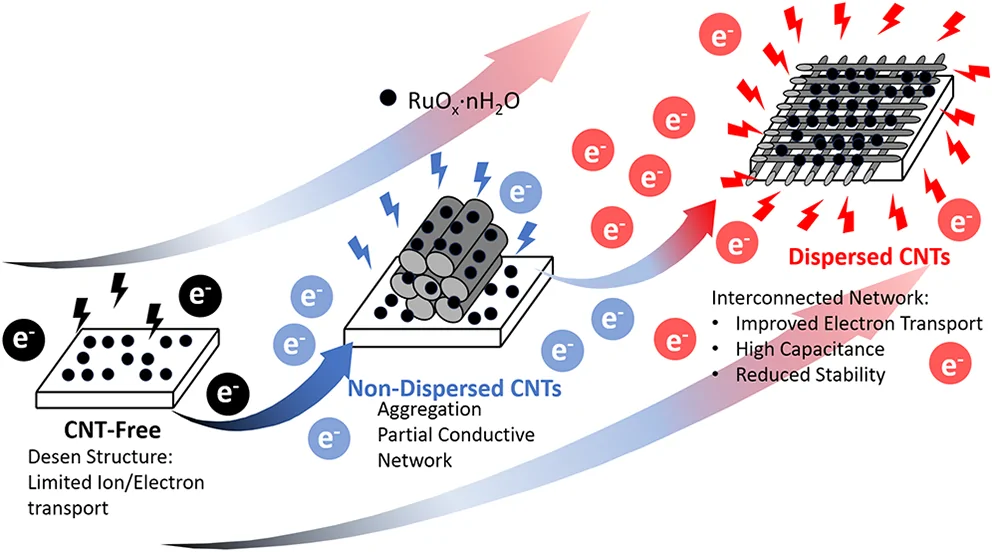

## 1 Introduction

Electrochemical supercapacitors have attracted extensive attention for high-power energy storage applications due to rapid charge–discharge capability, high power density, and long cycle life.^1–5^ Among various electrode materials, RuO*_x_* ·*n*H_2_O is widely recognized as a benchmark pseudocapacitive material because of its high theoretical capacitance and fast, reversible redox kinetics.^6–8^ The charge storage mechanism of RuO*_x_* ·*n*H_2_O is primarily governed by surface and near-surface redox reactions, which enable high-rate performance compared to conventional battery-type materials.^9–11^

Despite these advantages, the practical performance of RuO*_x_* ·*n*H_2_O-based electrodes remains limited by insufficient utilization of active material and inefficient electron and ion transport within the electrode structure. The intrinsically low conductivity of the oxide phase and the formation of dense or non-uniform coatings can hinder charge transport pathways, thereby reducing overall electrochemical efficiency.^12–16^ Consequently, structural engineering of the electrode architecture has become a critical strategy to improve performance.

To address these challenges, conductive carbon materials such as CNT have been incorporated as conductive additives. CNT offer high electrical conductivity, large specific surface area, and the ability to form interconnected conductive networks. These characteristics can facilitate electron transport and improve the accessibility of electrochemically active sites. However, CNT tend to aggregate due to strong van der Waals interactions, which significantly reduces their effective surface area and disrupts conductive pathways, leading to non-uniform electrode structures and inconsistent electrochemical performance.^17–21^

Therefore, electrode performance is strongly governed by various CNT conditions, particularly the dispersion state. Controlled dispersion of CNT can lead to distinct electrode architectures, ranging from compact structures to interconnected porous networks, which directly influence electron transport, ion diffusion, and active material utilization. However, while enhanced dispersion is generally associated with improved conductivity and capacitance, its impact on long-term structural stability remains less understood.^22–26^

In addition, the electrochemical behavior of hydrous RuO*_x_* ·*n*H_2_O is closely related to its structural characteristics, including hydration level, amorphous or nanocrystalline nature, and surface chemistry. The interplay between these intrinsic material properties and CNT incorporation introduces additional complexity to the electrode system.^27–32^ A systematic understanding of how dispersion-controlled CNT networks interact with hydrous RuO*_x_* ·*n*H_2_O during deposition and electrochemical cycling is still lacking.

As summarized in Table 1,^33–37^ numerous RuO_2_-based composite electrodes have been developed using porous carbon, graphene, CNT, MXene, and conductive polymers to improve electron transport and active-material utilization. Although these studies have demonstrated enhanced electrochemical performance, most efforts have focused on the selection of conductive supports, composite composition, or pore-structure engineering. Particularly, CNT-containing RuO_2_ electrodes have been widely reported owing to the excellent electrical conductivity and high specific surface area of CNT. However, previous studies generally treated CNT as conductive additives and rarely considered how the dispersion state of CNT influences the deposition behavior of RuO_2_, the formation of conductive networks, and the resulting structure–performance relationship. As a consequence, the fundamental role of CNT dispersion in governing charge transport, active-material utilization, and long-term cycling stability remains insufficiently understood.

**Table 1:** Comparison of RuO_2_/RuO*_x_*-Based Carbon Composite Electrodes for Supercapacitor Applications

electrode structure	electrolyte	specific capacitance	rate capability	cycling stability	key feature
RuO_2_/CAC^33^	3 M KOH	510 F g^–1^@1 A g^–1^	402 F g^–1^@20 A g^–1^ (78.8%)	87.1% after 3000 cycles	Microporous activated carbon + RuO_2_ nanoparticles
rHGO/CNT/RuO_2_ hydrogel^34^	37 wt % H_2_SO_4_	480 mF cm^–2^	83% retention at 20-fold current increase	93.9% after 10,000 cycles	3D hydrogel conductive framework
RuO_2_-MXene/CNT fibers^35^	1 M NaOH	181.6 F g^–1^		88.1% after 1000 cycles	Flexible fiber supercapacitor
PTh–PPy–RuO_2_ composite^36^	1 M H_2_SO_4_	820 F g^–1^@1 A g^–1^		89% after 3000 cycles	Conductive polymer-assisted pseudocapacitance
RuO_2_ Nanorods/CNF^37^	6 M KOH	188 F g^–1^	77–82% retention at 20 mA cm^–2^	93% after 3000 cycles	Surface area/mesopore/crystallinity optimization
RuO*_x_* ·*n*H_2_O/CNT (This Work)	0.5 M H_2_SO_4_	713.6 F g^–1^@5 mA cm^–2^	High capacitance retention at elevated current density, *b* ≈ 0.93	40% Evaluated up to 100,000 cycles	CNT dispersion engineering for enhanced RuO*_x_* utilization

To address this knowledge gap, this work systematically investigates the influence of CNT dispersion on the morphology, electrochemical behavior, and cycling durability of RuO*_x_* ·*n*H_2_O electrodes. By comparing CNT-free, non-dispersed CNT, and dispersed CNT systems, the present study aims to establish the structure–performance–stability relationship associated with CNT dispersion and provide insights for the rational design of pseudocapacitive electrodes.

In this study, RuO*_x_* ·*n*H_2_O/CNT composite electrodes are fabricated via a cathodic deposition approach with controlled CNT dispersion, enabling systematic modulation of electrode architecture. The effects of various CNT conditions and deposition parameters on electrode structure, charge transport behavior, and electrochemical performance are comprehensively investigated. We establish a structure–performance–stability relationship governed by dispersion-controlled electrode architecture and identify CNT dispersion as a key design parameter. These findings provide new insight into balancing conductivity and durability, offering a rational strategy for the design of high-performance pseudocapacitive systems. Unlike previous RuO_2_/CNT composite studies that primarily focused on composite fabrication and electrochemical performance, the present work systematically elucidates how CNT dispersion governs RuO*_x_* ·nH_2_O deposition, charge-transfer behavior, active-material utilization, and long-term cycling stability.^37,38^

## 2 Experiments

### 2.1 Materials

Ruthenium­(III) chloride hydrate (RuCl_3_·*x*H_2_O, 99.9%, Alfa) of 0.02M was used as the precursor for hydrous ruthenium oxide. A commercial multiwalled CNT (L-MWCNT1020, purity ≥97 vol %, amorphous carbon <3%, Conjutek Inc.) of 0.05 wt % was employed as a conductive additive and used as received. Ethanol of 20 wt % was used as a co-solvent, non-ionic surfactant (Triton X-100, Merck) of 0.02M as a surfactant, and potassium chloride (KCl, Sigma) 0.3M as a supporting electrolyte. Hydrochloric acid (HCl, Sigma) and potassium hydroxide (KOH, Sigma) aqueous solutions were used for pH adjustment. Titanium (Ti, 99.99%, Tai-mao) foil was used as the substrate. All chemicals were used as received.

### 2.2 Substrate Pretreatment

Ti substrates with a geometric area of 1 × 1 cm^2^ were used as current collectors. Prior to surface treatment, the substrates were sequentially cleaned in acetone, ethanol, and deionized water under ultrasonication to remove organic contaminants. To enhance surface roughness and interfacial adhesion, a two-step chemical etching process was applied. The Ti substrates were first treated in 5 wt % hydrofluoric acid (HF, Sigma) for 5 min to generate macro-void surface features. Subsequently, the substrates were immersed in 50 wt % HCl at 90 °C for 15 min to further develop tiny void surface structures.

After etching, the substrates were thoroughly rinsed with deionized water and dried under ambient conditions. This hierarchical surface modification improves interfacial contact between the substrate and the deposited active material, facilitating uniform coating and enhancing structural stability during electrochemical operation.

### 2.3 Electrolyte Preparation

The deposition solution was prepared by mixing deionized water with ethanol, followed by the addition of Triton X-100 to improve wettability and dispersion stability.^39–41^ The solution was subjected to ultrasonication (Autotune Series High Intensity Ultrasonic Processor, 750-Watt Model) for 30 mins to ensure homogeneous mixing. CNT were introduced into the solution under various dispersion conditions. For the non-dispersed condition, CNT were directly added without further treatment. For the dispersed condition, CNT were ultrasonically treated using a high-power ultrasonic processor to achieve uniform dispersion. The presence of Triton X-100 further facilitated dispersion by reducing surface tension and preventing CNT agglomeration. The dispersion state of CNT was controlled as a key experimental variable to investigate its effect on electrode structure and electrochemical performance. RuCl_3_·*x*H_2_O was then dissolved in the prepared solution, followed by the addition of KCl as a supporting electrolyte to enhance ionic conductivity. The suspension was continuously stirred to maintain a uniform distribution of CNT during deposition. The solution was protected from light to prevent undesired reduction of ruthenium ions and maintained under constant stirring for 8 h before use.

### 2.4 Cathodic Deposition of RuO*_x_* ·*n*H_2_O

Cathodic deposition was carried out using a galvanostatic method. The pretreated Ti substrate was used as the working electrode, and a platinum plate (Pt) was used as the counter electrode.^41–43^ Before and after deposition, the substrate should be dried. And the substrate was weighed by the precision electronic microbalance (Sartorios, MC-5). Deposition was conducted at a current density of approximately 10 mA cm^–2^ under continuous stirring to ensure uniform ion transport. The deposition time was varied from 5 to 60 min to investigate the effect of growth duration on electrode structure and performance.

During the cathodic process, the Ti electrode surface underwent reduction reactions, leading to the formation and deposition of hydrous ruthenium oxide (RuO*_x_* ·*n*H_2_O) on the substrate. After deposition, the electrodes were rinsed with deionized water to remove residual electrolyte and dried at room temperature. The active mass loading of RuO*_x_* ·*n*H_2_O was determined from the weight difference of the electrode before and after deposition. The average loading was approximately 0.630 mg cm^–2^ for the CNT-free electrode, 0.827 mg cm^–2^ for the non-dispersed CNT electrode, and 1.330 mg cm^–2^ for the dispersed CNT electrode, as shown in Table S1.

### 2.5 Structural and Chemical Characterization

Surface morphology of the electrodes was examined using scanning electron microscopy (SEM). Transmission electron microscopy (TEM) was employed to investigate nanoscale structure and structural evolution before and after electrochemical cycling. Chemical composition and oxidation states were analyzed using X-ray photoelectron spectroscopy (XPS), with particular focus on the Ru 3d and O 1s regions to identify the oxidation state of ruthenium and the chemical environment of oxygen species.^44–46^

These characterization techniques provide complementary insights into morphology and chemical states, forming the basis for understanding the structure–performance relationship discussed in subsequent sections.

### 2.6 Electrochemical Measurements

Electrochemical performance was evaluated using a potentiostat/galvanostat (AUTOLAB, PGSTAT12) in a standard three-electrode configuration. The prepared electrode was used as the working electrode, a saturated calomel electrode (SCE, Hg/Hg_2_Cl_2_ in saturated KCl) served as the reference electrode, and a Pt plate was used as the counter electrode. A H_2_SO_4(aq)_ of 0.5M was used as the electrolyte.^47–49^

Cyclic voltammetry (CV) measurements were conducted within a potential window of 0–1 V at a scan rate of 5 mV s^–1^. For an ideal capacitor, the CV curve typically exhibits a rectangular shape, indicating a constant capacitance response. In the present system, the RuO*_x_* ·*n*H_2_O electrode undergoes continuous surface redox reactions during the potential sweep, resulting in quasi-rectangular profiles characteristic of pseudocapacitive behavior.^50–53^

The capacitance is defined as

1 C=Q/V

where *C* is the capacitance, *Q* is the stored charge, and *V* is the applied potential. Under dynamic CV conditions, the capacitance can be expressed as

2 C=(dQ/dt)/(dV/dt)=I/υ

where *I* is the current and υ is the scan rate.

For standard electrochemical characterization (CV and GCD), the capacitance is reported as gravimetric capacitance (F g^–1^), calculated based on the mass of the active material. Galvanostatic charge–discharge (GCD) measurements were performed at a current density of 5 mA cm^–2^ to determine capacitance and cycling performance. Electrochemical impedance spectroscopy (EIS) measurements were carried out over a wide frequency range to analyze charge-transfer resistance and ion diffusion behavior. All measurements were conducted at room temperature under ambient conditions.

For long-term cycling tests up to 100,000 cycles, the mass of the active material could not be reliably determined without interrupting the measurement. Therefore, the electrochemical performance under these conditions is reported in terms of areal capacitance (F cm^–2^), which provides a more appropriate evaluation for continuous cycling.

## 3 Results and Discussion

Figure 1 presents a conceptual illustration of CNT dispersion–controlled RuO*_x_* ·*n*H_2_O electrode architecture. The dispersion state of CNT governs the formation of distinct electrode configurations, ranging from compact structures to interconnected conductive networks. These structural variations are expected to influence electron transport, ion accessibility, and active material distribution. This schematic provides a conceptual framework for interpreting the morphological evolution and electrochemical behavior discussed in the following sections.

**Figure 1:**
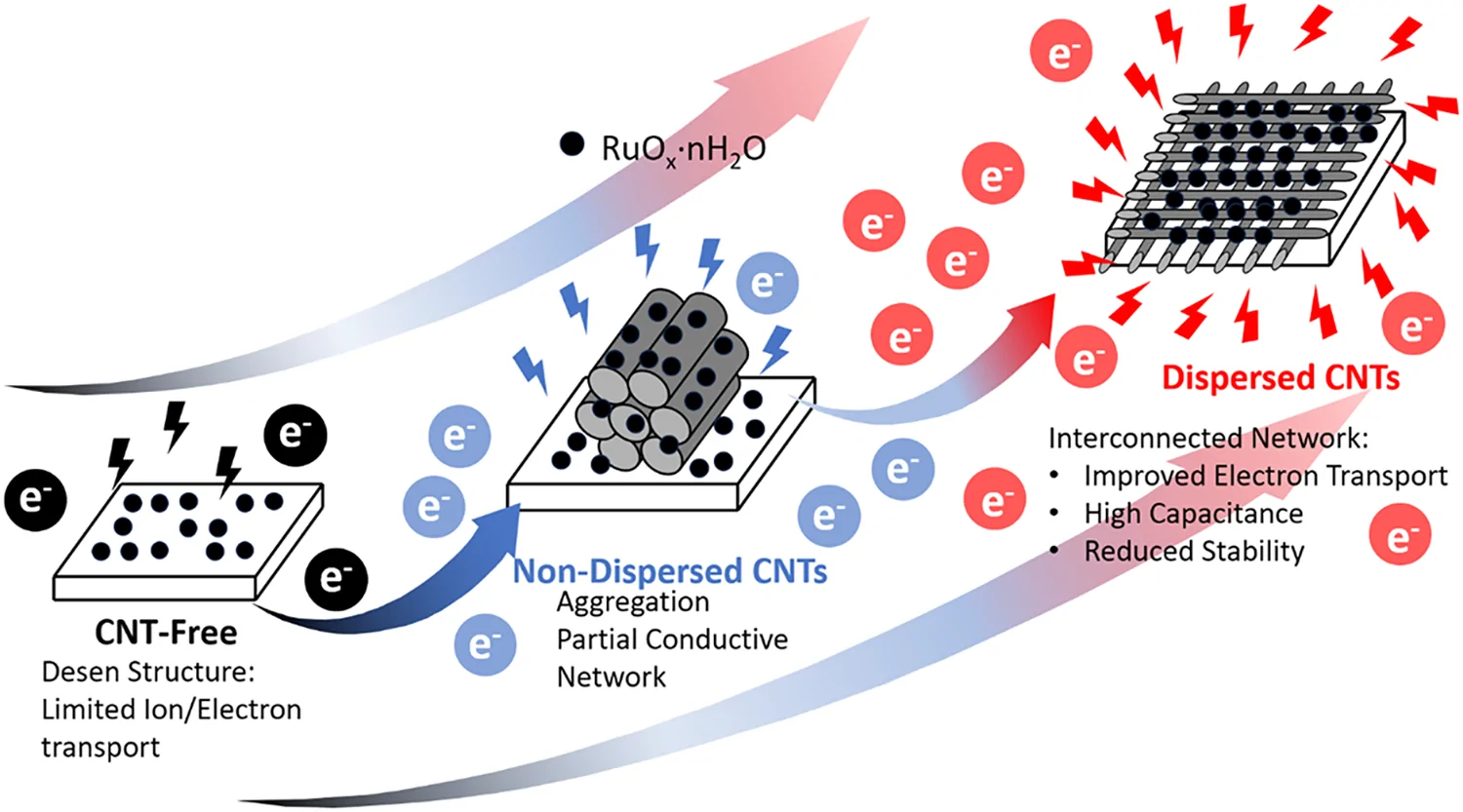
Conceptual illustration of CNT dispersion–controlled RuO*_x_* ·*n*H_2_O electrode architecture. CNT-free electrodes exhibit dense structures with limited transport. Non-dispersed CNT lead to aggregation and partial conductive pathways. Dispersed CNT form interconnected networks that enhance charge transport and capacitance, but reduce structural stability.

### 3.1 Morphological Evolution under Various CNT Conditions

The formation of hydrous RuO*_x_* ·*n*H_2_O during cathodic deposition can be described by a series of electrochemical reactions. Initially, Ru^3+^ ions are reduced to metallic Ru (eq 3), accompanied by hydrogen evolution and hydroxide generation (eq 4). Subsequently, the deposited Ru reacts with hydroxide ions to form intermediate Ru oxyhydroxide species (eq 5), which further transform into hydrous RuO*_x_* ·*n*H_2_O through hydration and oxidation processes (eq 6).

3 $${\mathrm{Ru}}^{3+}+3e^{-}\to \mathrm{Ru}$$

4 $$H_{2}O+e^{-}\to {\mathrm{OH}}^{-}+1/2H_{2}↑$$

5 $$\mathrm{Ru}+\left(x+1\right){\mathrm{OH}}^{-}\to {\mathrm{RuO}\left(\mathrm{OH}\right)}_{x}+\left(x+1\right)e^{-}$$

6 $${\mathrm{RuO}\left(\mathrm{OH}\right)}_{x}+nH_{2}O\to {\mathrm{RuO}}_{1+x}\cdot nH_{2}O+x/2H_{2}↑$$

These reactions lead to the formation of hydrous, amorphous RuO*_x_* ·*n*H_2_O, which is consistent with the XPS results discussed in Section 3.2.

Based on the controlled CNT dispersion conditions introduced during electrolyte preparation, the resulting electrode morphology was systematically examined using scanning electron microscopy (SEM), as shown in Figure 2. The structural features observed reflect the influence of various CNT conditions on the nucleation and growth behavior of RuO*_x_* ·*n*H_2_O during deposition.

**Figure 2:**
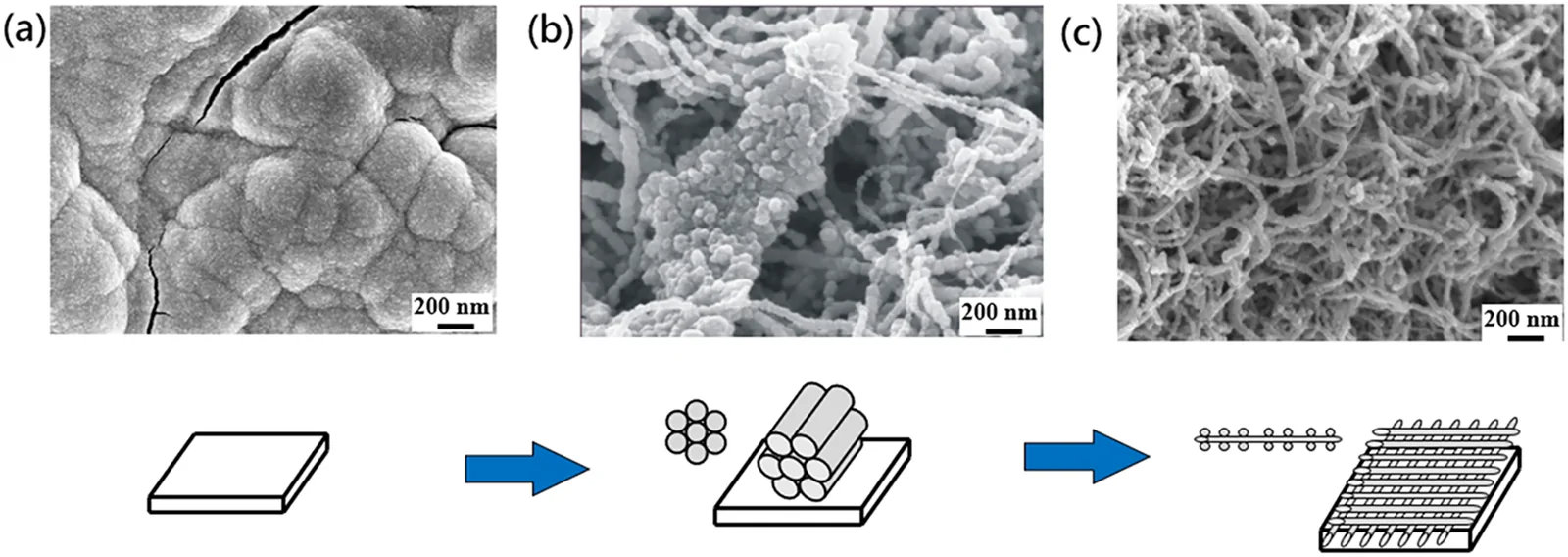
SEM images of RuO*_x_* ·*n*H_2_O electrodes prepared under various CNT conditions: (a) CNT-free, (b) non-dispersed CNT, and (c) dispersed CNT. The CNT-free electrode shows a dense RuO*_x_* ·*n*H_2_O morphology, while non-dispersed CNT result in aggregation. Dispersed CNT promote a more uniform and interconnected RuO*_x_* ·*n*H_2_O structure.

As shown in Figure 2(a), the CNT-free electrode exhibits a dense and compact morphology with limited porosity. Such a structure is attributed to the direct deposition of RuO*_x_* ·*n*H_2_O without conductive scaffolding, resulting in restricted electrolyte penetration and limited active surface exposure. In contrast, Figure 2(b) shows that the addition of CNT without dispersion leads to significant aggregation, forming non-uniform clusters embedded within the RuO*_x_* ·*n*H_2_O matrix. This aggregated structure disrupts the continuity of conductive pathways and results in heterogeneous coating, which is expected to negatively affect both electron transport and electrochemical utilization.

When CNT are well dispersed, as shown in Figure 2(c), a highly interconnected network structure is formed. The uniformly distributed CNT act as conductive frameworks, facilitating the deposition of RuO*_x_* ·*n*H_2_O into a more porous and homogeneous architecture. This structure enhances electrolyte accessibility and promotes efficient charge transport throughout the electrode. The non-dispersed CNT specimen exhibits noticeable CNT bundles and localized aggregation, whereas the dispersed CNT specimen displays a more homogeneous and interconnected network structure. These observations confirm that the ultrasonication-assisted dispersion treatment effectively reduces CNT aggregation and promotes a more uniform distribution of CNT within the electrode architecture.

The structural differences observed under various CNT conditions directly reflect the effectiveness of dispersion control during the deposition process. In particular, the dispersed CNT network not only modifies the morphology of the active material but also establishes continuous electron transport pathways, which are critical for improving electrochemical performance. However, the formation of a thinner and more distributed RuO*_x_* ·*n*H_2_O coating on CNT frameworks may introduce potential structural instability, which will be further discussed in subsequent sections.

### 3.2 Chemical State Analysis

The chemical composition and oxidation states of the deposited RuO*_x_* ·*n*H_2_O were analyzed by X-ray photoelectron spectroscopy (XPS), as shown in Figure 3(a). The high-resolution Ru 3d spectrum (Figure 3(b)) shows two main peaks located at 280.94 and 285.11 eV, corresponding to Ru 3d_5/2_ and Ru 3d_3/2_, respectively. These binding energies are consistent with oxidized Ru species and indicate that Ru^4+^ is the dominant chemical state in the deposited ruthenium oxide. The fitted peak area ratio of the Ru 3d_5/2_ to Ru 3d_3/2_ components was approximately 1:0.83, consistent with the spin–orbit doublet of oxidized ruthenium. These binding energies confirm that Ru^4+^ is the dominant oxidation state in the deposited hydrous RuO*_x_* ·*n*H_2_O rather than metallic Ru.^54–57^

**Figure 3:**
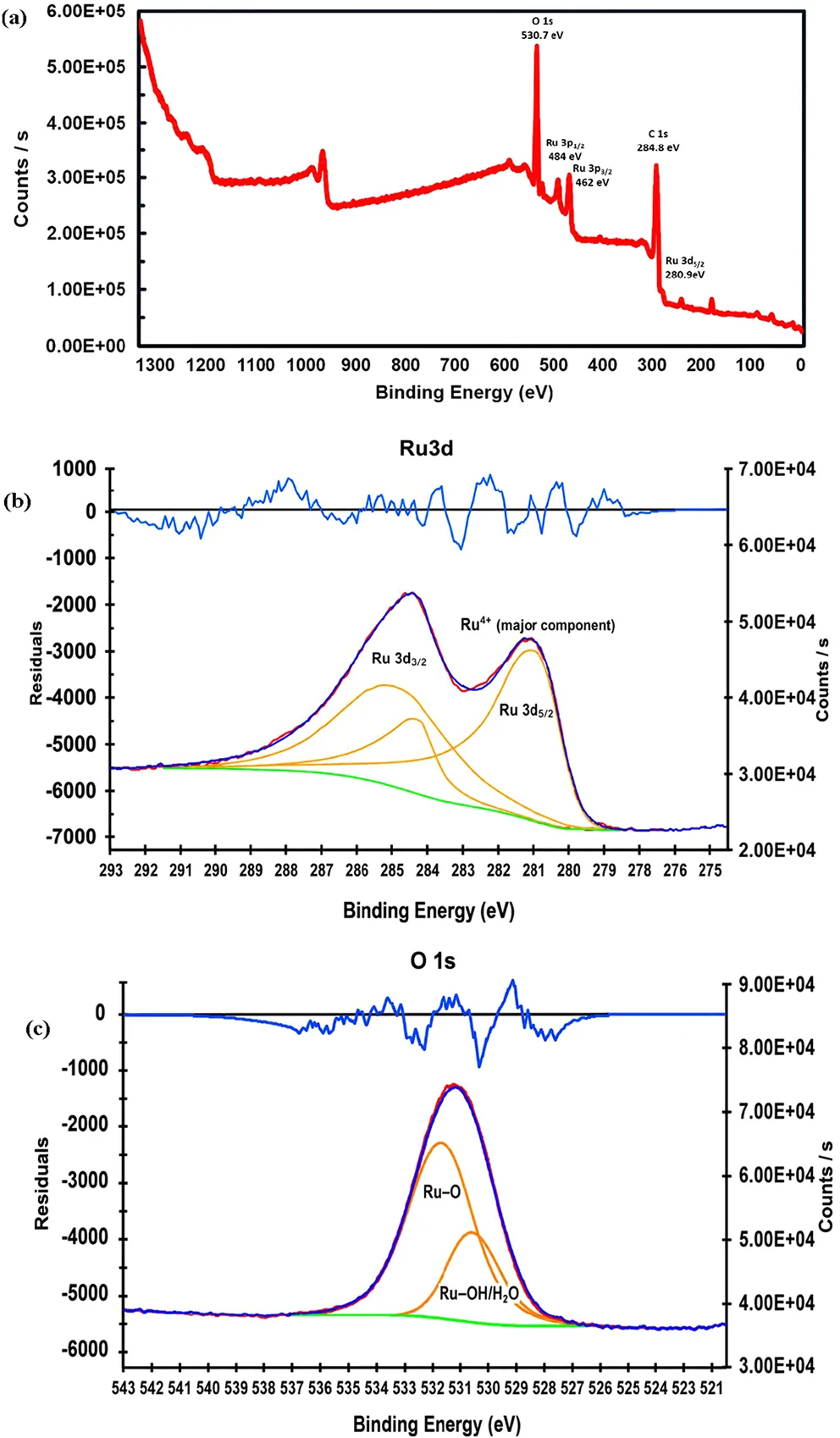
XPS spectra of RuO*_x_* ·*n*H_2_O electrodes: (a) The survey spectrum (b) Ru 3d and (c) O 1s regions. The spectra indicate the presence of oxidized Ru species and hydroxyl/water components, confirming the formation of hydrous RuO*_x_* ·*n*H_2_O.

The O 1s spectrum (Figure 3(c)) was deconvoluted into two components. The peak centered at 530.70 eV is assigned to lattice oxygen (Ru–O), whereas the peak at 531.69 eV corresponds to surface hydroxyl groups and adsorbed water species (Ru–OH/H_2_O). The fitted area ratio of Ru–O to Ru–OH/H_2_O is approximately 1:0.41, indicating that a substantial fraction of oxygen is associated with hydroxylated and hydrated environments. The coexistence of lattice oxygen and hydroxyl/water-related species confirms the formation of hydrous RuO*_x_* ·*n*H_2_O rather than fully crystalline stoichiometric RuO_2_. These hydrated oxygen species are considered to facilitate proton-coupled surface redox reactions and contribute to the pseudocapacitive behavior of the electrode.The coexistence of Ru–O and Ru–OH/H_2_O species is important for pseudocapacitive charge storage. The Ru–O framework provides redox-active ruthenium oxide sites, while hydroxyl and adsorbed water species facilitate proton transport and reversible surface redox reactions. Therefore, the XPS results support the formation of hydrous RuO*_x_* ·*n*H_2_O with abundant oxygen-containing species, which contributes to the observed pseudocapacitive behavior and high capacitance.

### 3.3 Electrochemical Performance under Various CNT Conditions

The electrochemical performance of the electrodes under various CNT conditions was evaluated using cyclic voltammetry (CV) and galvanostatic charge–discharge (GCD), as shown in Figure 4.

**Figure 4:**
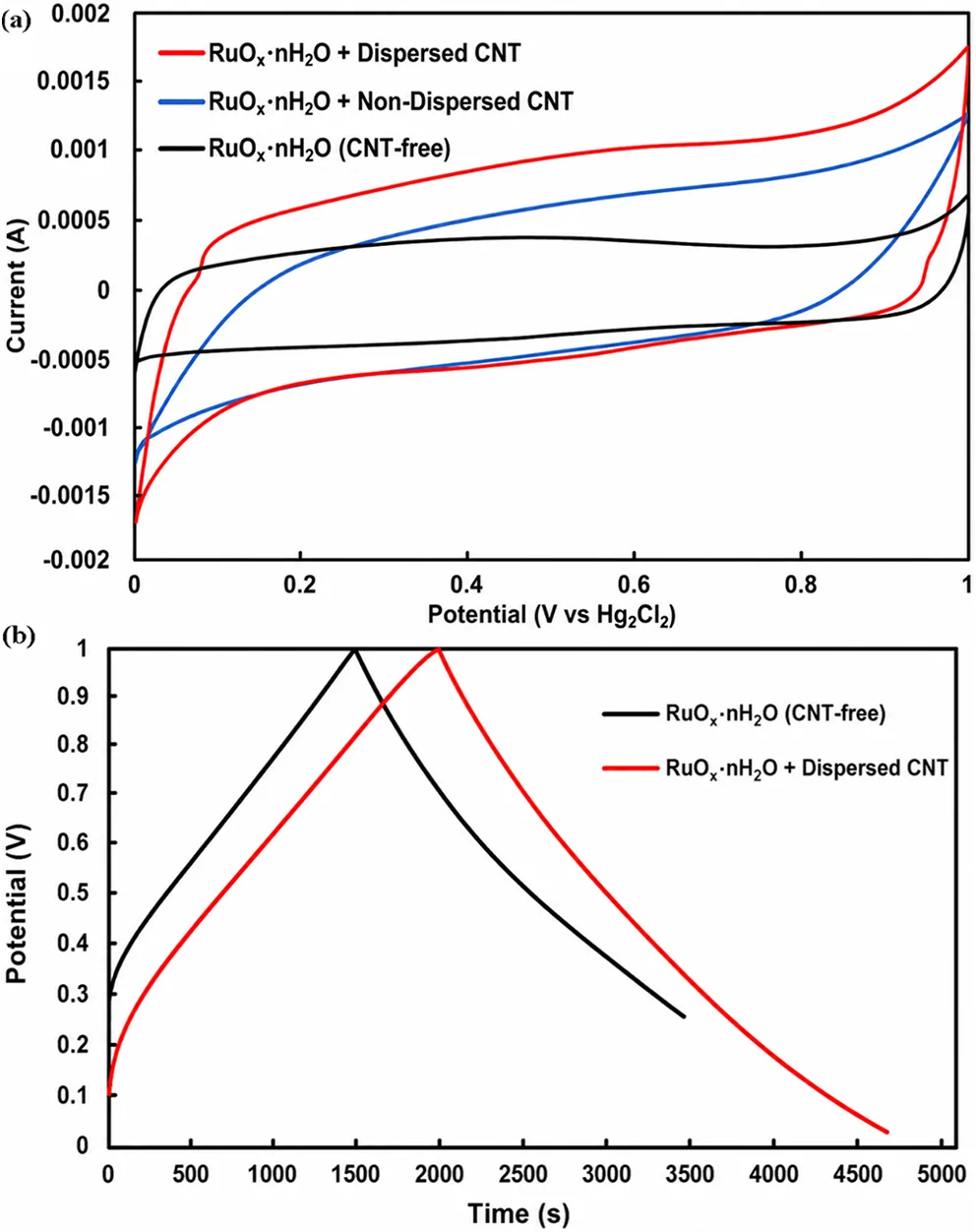
Electrochemical performance of RuO*_x_* ·*n*H_2_O electrodes under various CNT conditions: (a) CV curves at 5 mV s^–1^ and (b) GCD profiles at 5 mA cm^–2^. Dispersed CNT significantly enhance the charge storage behavior of RuO*_x_* ·*n*H_2_O, resulting in higher current response and longer discharge time.

As shown in Figure 4(a), the CV curves exhibit quasi-rectangular shapes, indicating typical capacitive behavior associated with rapid surface redox reactions. The dispersed CNT electrode shows a significantly higher current response compared to the CNT-free electrode, suggesting enhanced charge storage capability.

Figure 4(b) shows the GCD curves, where the electrode with dispersed CNT exhibits a longer discharge time and improved curve symmetry, indicating higher capacitance and reduced internal resistance. The calculated capacitance values increase from 428.0 F g^–1^ for the CNT-free electrode to 590.0 F g^–1^ for the non-dispersed CNT electrode, and further to 713.6 F g^–1^ for the dispersed CNT electrode. The GCD profile of the RuO*_x_* ·nH_2_O electrode with non-dispersed CNT is provided in Figure S1 to further verify the influence of CNT dispersion on capacitance.

These improvements can be attributed to the interconnected conductive network formed by dispersed CNT, which enhances electron transport and increases electrochemically active surface area. The observed performance enhancement confirms that CNT dispersion plays a key role in governing electrochemical behavior, thereby necessitating further optimization of CNT content and deposition conditions.

### 3.4 Optimization of Dispersed CNT Content and Deposition Time (NEW)

To identify the optimal fabrication parameters, the effects of dispersed CNT content and deposition time on the electrochemical performance of RuO*_x_* ·nH_2_O electrodes were systematically investigated. The corresponding capacitance values are summarized in Figure 5, while the associated CV curves are provided in Figure S2. Additional CV measurements recorded at scan rates ranging from 5 to 50 mV s^–1^ are provided in Figure S3.^58,59^ The preservation of the overall CV shape at increasing scan rates indicates favorable charge-transfer kinetics and pseudocapacitive behavior.

**Figure 5:**
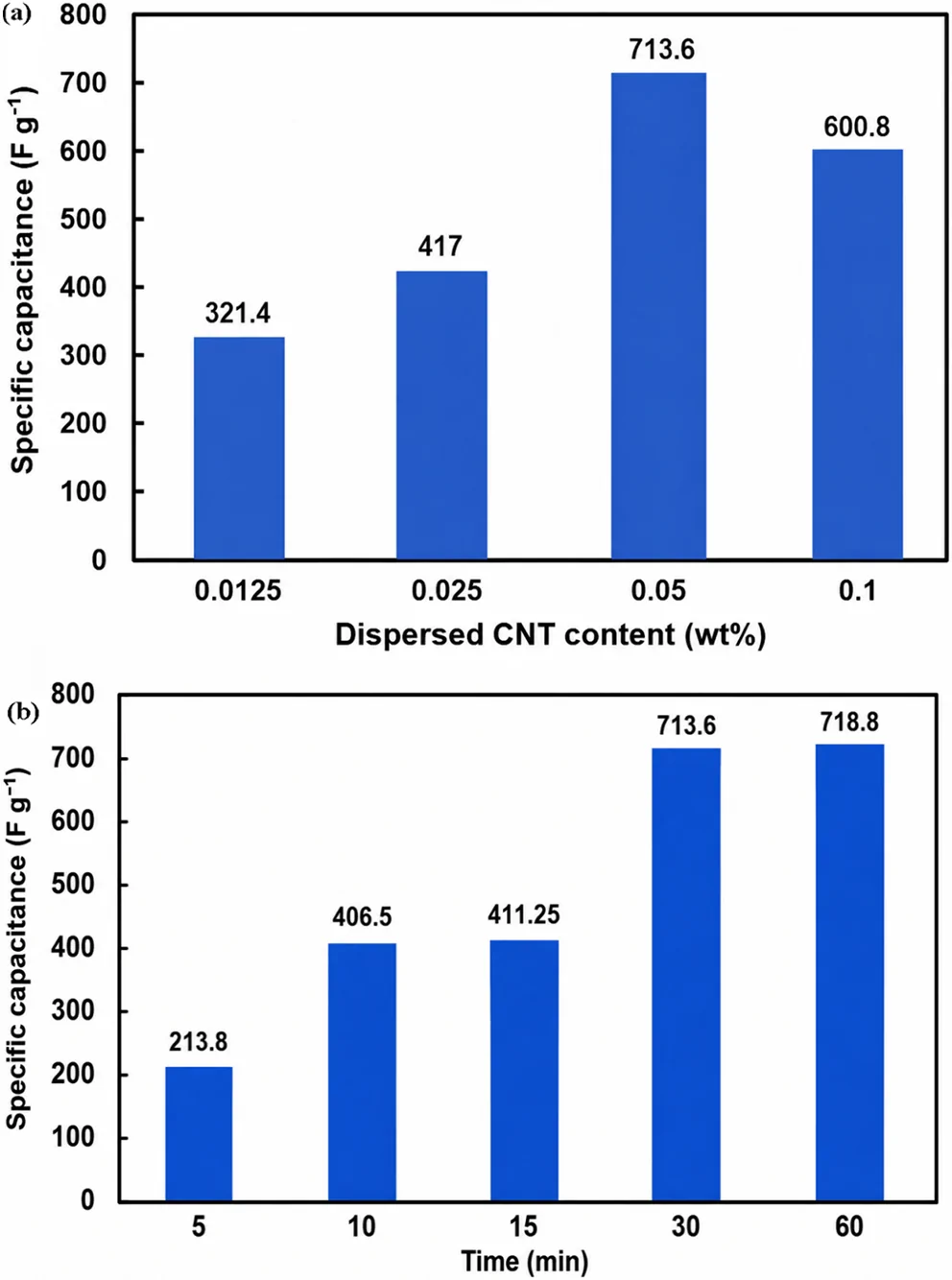
Optimization of RuO*_x_* ·nH_2_O electrochemical performance: (a) specific capacitance as a function of dispersed CNT content (wt %) and (b) specific capacitance as a function of deposition time (min). An optimal condition is observed at intermediate dispersed CNT loading and deposition time.

As shown in Figure 5(a), the specific capacitance increases with increasing dispersed CNT content and reaches a maximum value of 713.6 F g^–1^ at 0.05 wt %. The enhanced capacitance can be attributed to the formation of a more effective conductive network, which improves electron transport and facilitates electrochemical utilization of the RuO*_x_* ·*n*H_2_O active material. In addition, the improved dispersion of CNT promotes a more uniform distribution of RuO*_x_* ·*n*H_2_O, increasing the accessibility of electrochemically active sites. However, when the CNT content is further increased to 0.10 wt %, the capacitance decreases. This behavior is likely associated with CNT aggregation, which disrupts the conductive network and leads to non-uniform deposition of the active material, thereby reducing its utilization efficiency.

The influence of deposition time is shown in Figure 5(b). The specific capacitance increases continuously as the deposition time is extended from 5 to 30 min, reaching a maximum value of 713.6 F g^–1^ at 30 min. The increase in capacitance suggests that longer deposition times provide a greater amount of electrochemically active RuO*_x_* ·*n*H_2_O on the electrode surface. However, extending the deposition time beyond 30 min yields only marginal improvement. Excessive deposition may result in a thicker oxide layer, which increases ion-transport resistance and limits the accessibility of inner active sites, thereby reducing the effectiveness of additional active-material loading.

These results indicate that the electrochemical performance of RuO*_x_* ·*n*H_2_O electrodes is governed by a balance between conductive-network formation and active-material accessibility. Optimized CNT dispersion enhances charge transport and active-site utilization, whereas excessive CNT loading or oxide deposition can hinder efficient electrochemical utilization.

To further investigate the charge-storage mechanism, a *b*-value analysis based on CV measurements at different scan rates was performed (Figure S3). A *b*-value of approximately 0.93 was obtained, indicating that the charge-storage process is predominantly surface-controlled.^60,61^ This result suggests that the enhanced performance achieved at the optimized CNT content and deposition time originates primarily from improved electrochemical accessibility and charge transport rather than diffusion-limited bulk storage.

Overall, the optimization results demonstrate that the electrochemical performance of RuO*_x_* ·*n*H_2_O electrodes is governed by a balance between conductive network formation, active-material utilization, and ion-transport accessibility. The optimum condition was achieved at 0.05 wt % dispersed CNT and a deposition time of 30 min.

### 3.5 Charge Transport Behavior (EIS)

Electrochemical impedance spectroscopy (EIS) measurements were performed over a frequency range of 100 kHz to 0.01 Hz using an AC perturbation amplitude of 10 mV at a fixed applied potential of 0.1 V vs Ag/AgCl to investigate the charge-transfer characteristics of the electrodes, as shown in Figure 6. The Nyquist plots exhibit a depressed semicircle in the high-frequency region followed by an inclined line in the low-frequency region, corresponding to charge-transfer and ion-transport processes, respectively. Since the primary objective of the EIS measurements was to qualitatively compare the electrochemical characteristics of different electrodes, equivalent circuit fitting was not performed. Instead, the solution resistance (*R*_s_) and charge-transfer resistance (*R*_ct_) were estimated from the high-frequency intercept and the diameter of the semicircle in the Nyquist plots, respectively, and are summarized in Table S3.^62–64^ The dispersed CNT electrode exhibits the smallest semicircle diameter among all specimens, with the estimated Rct decreasing from approximately 1.50 Ω for the CNT-free RuO*_x_* ·*n*H_2_O electrode to 0.90 Ω after CNT dispersion, indicating enhanced interfacial charge-transfer kinetics. This result is consistent with the formation of a more continuous conductive network after CNT dispersion, which facilitates electron transport across the electrode/electrolyte interface.

**Figure 6:**
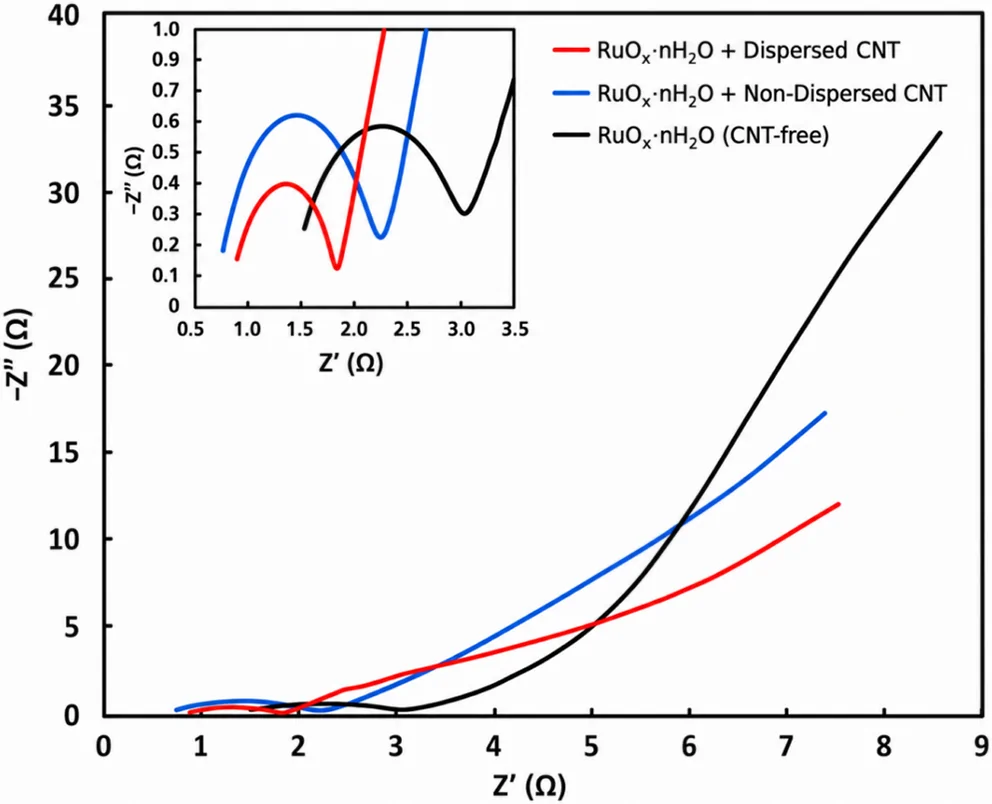
Nyquist plots of RuO*_x_* ·*n*H_2_O electrodes prepared under various CNT conditions.

Additional EIS measurements for various dispersed CNT contents and various deposition times have been included in the supporting information (Figures S4). Furthermore, the dispersed CNT electrode exhibits a more favorable low-frequency impedance response, suggesting improved electrolyte accessibility and ion transport within the CNT-supported RuO*_x_* ·nH_2_O structure. These improvements can be attributed to the formation of an interconnected CNT network, which facilitates both electron transport and electrolyte penetration throughout the electrode architecture.

The reduced charge-transfer resistance and enhanced ion-transport behavior are consistent with the electrochemical performance results discussed in Sections 3.3 and 3.4. Together, these findings demonstrate that CNT dispersion plays a critical role in regulating electron- and ion-transport pathways, thereby contributing to the enhanced pseudocapacitive performance of RuO*_x_* ·nH_2_O electrodes.

### 3.6 Cycling Stability and Structure–Performance Trade-Off

The long-term cycling stability of RuO*_x_* ·*n*H_2_O electrodes, with and without dispersed CNT, was evaluated by repeated galvanostatic charge–discharge measurements, as shown in Figure 7. The CNT-free RuO*_x_* ·*n*H_2_O electrode exhibits excellent stability, maintaining nearly 100% capacitance retention over 100,000 cycles, indicating robust structural integrity and strong interfacial adhesion of the active material. In contrast, the RuO*_x_* ·*n*H_2_O electrode incorporating dispersed CNT delivers a higher initial capacitance but undergoes significant degradation during prolonged cycling, retaining only approximately 40% of its initial capacitance. This pronounced contrast highlights a clear trade-off between electrochemical performance and structural stability. The enhanced initial capacitance in the dispersed CNT system originates from the increased electrochemical accessibility and improved charge transport provided by the interconnected CNT network, as discussed in Sections 3.3–3.5. The high-surface-area CNT framework promotes the distribution of RuO*_x_* ·*n*H_2_O over a larger interfacial area, resulting in greater utilization of the pseudocapacitive active material during the initial cycling stage.

**Figure 7:**
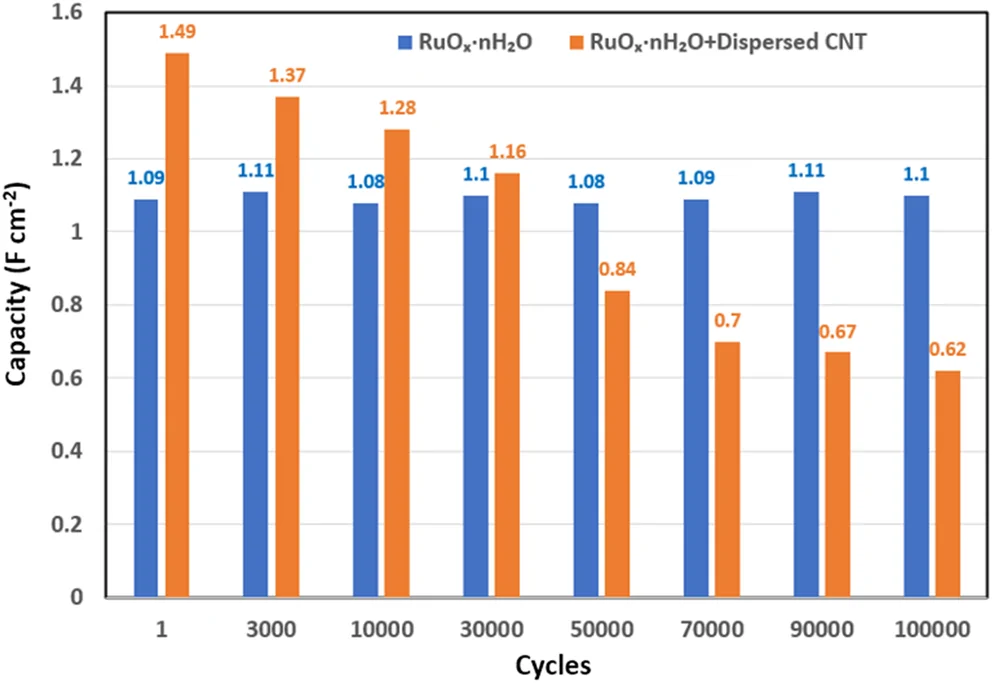
Cycling stability of RuO*_x_* ·*n*H_2_O electrodes (expressed as area capacitance, F cm^–2^) with and without dispersed CNT over 100,000 charge–discharge cycles.

However, such a highly dispersed architecture may also be more susceptible to structural degradation during prolonged redox cycling. Repeated charge–discharge processes can induce local volume changes and interfacial stress within the hydrated RuO*_x_* ·*n*H_2_O coating, potentially leading to a gradual reduction in electrochemically active sites and capacitance retention. Therefore, while CNT dispersion significantly enhances the initial electrochemical performance, it also introduces a performance–stability trade-off that must be considered in electrode design. This interpretation is qualitatively supported by the post-cycling TEM observations shown in Figure 8.

**Figure 8:**
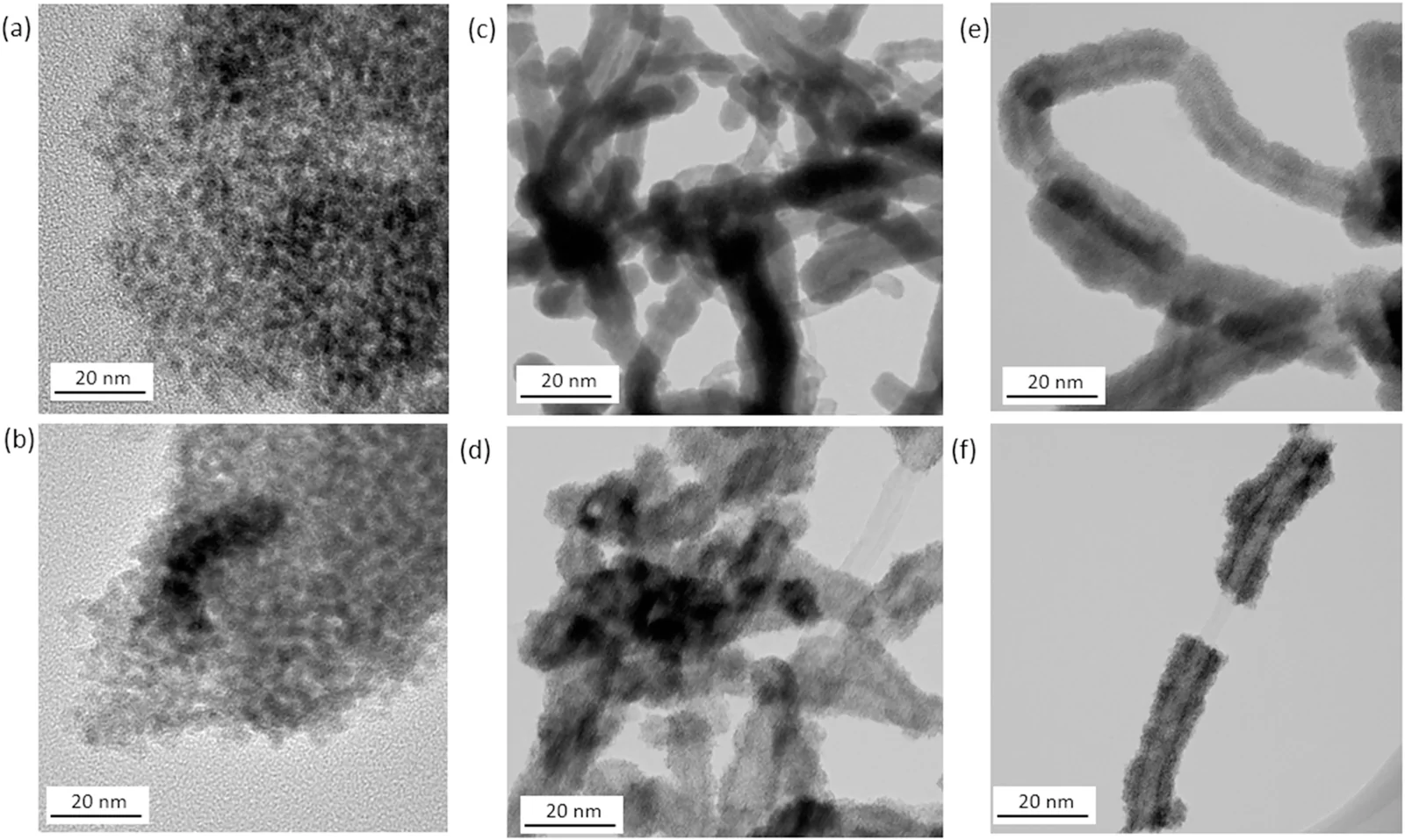
TEM images showing the structural evolution of RuO*_x_* ·nH_2_O electrodes before and after 100,000 charge–discharge cycles, (a) CNT-free electrode before cycling, (b) CNT-free electrode after cycling; (c) non-dispersed CNT electrode before cycling; (d) non-dispersed CNT electrode after cycling; (e) dispersed CNT electrode before cycling; (f) dispersed CNT electrode after cycling.

### 3.7 Structural Evolution during Long-Term Cycling

To further elucidate the origin of the observed cycling behavior, TEM was employed to examine the structural evolution of the electrodes before and after long-term cycling, as shown in Figure 8. The CNT-free RuO*_x_* ·*n*H_2_O electrode exhibits a relatively compact and continuous morphology before cycling (Figure 8(a)). After 100,000 cycles, the overall structure remains largely intact with only minor morphological changes (Figure 8(b)), suggesting relatively good structural stability. This observation is consistent with the nearly 100% capacitance retention shown in Figure 7 and suggests that the densely deposited RuO*_x_* ·*n*H_2_O layer may possesses strong interfacial adhesion and resistance to repeated electrochemical stress.

The electrode containing non-dispersed CNT initially exhibits aggregated CNT bundles coated with RuO*_x_* ·*n*H_2_O (Figure 8(c)). After prolonged cycling, localized structural disruption and partial collapse of the aggregated regions are observed (Figure 8(d)). Although the CNT network provides additional conductive pathways, the non-uniform distribution of CNT limits effective utilization of active material. The non-uniform CNT distribution may limit active-material utilization and contribute to localized degradation during cycling.

In contrast, the dispersed CNT electrode displays a homogeneous CNT network with a uniformly distributed RuO*_x_* ·*n*H_2_O coating prior to cycling (Figure 8(e)). This morphology is beneficial for electron transport and active-site accessibility, which explains the enhanced capacitance (713.6 F g^–1^) and reduced charge-transfer resistance observed in Sections 3.4 and 3.5. However, after 100,000 cycles, the RuO*_x_* ·nH_2_O coating becomes fragmented and discontinuous (Figure 8(f)), suggesting partial detachment or redistribution of the active material after prolonged cycling. The highly distributed RuO*_x_* ·*n*H_2_O layer appears to be more susceptible to repeated redox-induced volume changes, which may result in progressive loss of electrochemically active material.

These observations provide qualitative morphological evidence supporting the observed trade-off between electrochemical performance and cycling stability. The TEM observations, together with the electrochemical results, suggest that CNT dispersion promotes the formation of an interconnected conductive network and improves active-material utilization, leading to enhanced capacitance and charge-transfer characteristics. However, the thinner and more distributed RuO*_x_* ·*n*H_2_O coating formed on the CNT framework exhibits reduced long-term structural robustness. Taken together with the electrochemical results, the TEM observations support a structure–performance–stability relationship, suggesting that CNT dispersion is an important factor governing both the electrochemical efficiency and the long-term cycling behavior of RuO*_x_* ·*n*H_2_O electrodes.

## 4 Conclusions

In summary, hydrous RuO*_x_* ·*n*H_2_O electrodes with controlled CNT dispersion were successfully fabricated to elucidate the influence of CNT dispersion on the structure–performance relationship of pseudocapacitive electrodes. The results show that CNT dispersion significantly influences the morphology, charge-transfer characteristics, and electrochemical utilization of RuO*_x_* ·*n*H_2_O. Under the optimized conditions, the dispersed CNT electrode achieved a high specific capacitance of 713.6 F g^–1^, accompanied by reduced charge-transfer resistance and improved ion accessibility.

Long-term cycling revealed a clear trade-off between electrochemical performance and structural stability. The CNT-free RuO*_x_* ·*n*H_2_O electrode maintained nearly 100% capacitance retention after 100,000 cycles, whereas the dispersed CNT electrode retained approximately 40% of its initial capacitance. TEM observations suggest that the uniformly distributed RuO*_x_* ·*n*H_2_O coating on the CNT network, although highly effective for charge storage, became more susceptible to structural degradation during prolonged redox cycling, possibly owing to its thinner and more highly dispersed morphology.

Overall, this study demonstrates a clear correlation between CNT dispersion and the structure–performance–stability relationship of RuO*_x_* ·*n*H_2_O electrodes. Rather than simply serving as conductive additives, dispersed CNT play an active role in regulating RuO*_x_* ·*n*H_2_O deposition, charge transport, active-material utilization, and long-term cycling behavior. These findings suggest that engineering CNT dispersion is an effective strategy for optimizing pseudocapacitive electrodes and provides valuable design guidelines for the development of next-generation RuO*_x_*-based energy-storage materials.

## Supplementary Material



## Notes

Additional electrochemical characterization, including GCD profiles, CV curves obtained at different CNT contents and deposition times, and b-value analysis; morphological and XRD characterization; active-material loading; XPS fitting parameters; and estimated solution and charge-transfer resistance values derived from Nyquist plot analysis (Figures S1–S5 and Tables S1–S3) (PDF)
