# Supplementary material for: Dispersion-Controlled Carbon Nanotube Networks Enable Tunable Structure–Performance–Stability Relationships in RuO x ·nH2O Pseudocapacitive Electrodes
Source: ACS Omega. 2026 Sep 14;11(38):57172–82. doi: 10.1021/acsomega.6c05011 (PMC13629388; doi:10.1021/acsomega.6c05011)
Supplement: Supplementary file 1 [file ao6c05011_si_001.pdf]

Dispersion-Controlled Carbon Nanotube Networks Enable Tunable Structure–Performance–Stability Relationships in RuOx·nH2O Pseudocapacitive Electrodes

Wilson Hou-Sheng Huang[1, 2, *], *Yuli Lin*[2], *and Wen-Jin Li*[3]

[1]Center for Continuing Education, National Tsing Hua University, No.101, Section 2, Kuang-Fu Rd., Hsinchu City, 300044, Taiwan (R.O.C.)

[2]Department of Opto-Electronics and Material Engineering, Chung Hua University, 707, Sec. 2, WuFu Rd., Hsinchu City, 30012, Taiwan, (R.O.C.)

[3]Material and Chemical Research Laboratories, Industrial Technology Research Institute, No. 195, Sec. 4, Zhongxing Rd., Zhudong Township, Hsinchu County, 310401, Taiwan (R.O.C.)

A comparison including the non-dispersed CNT electrode is provided in Figure S1, confirming that CNT addition improves capacitance and that further dispersion enhances charge-storage performance.

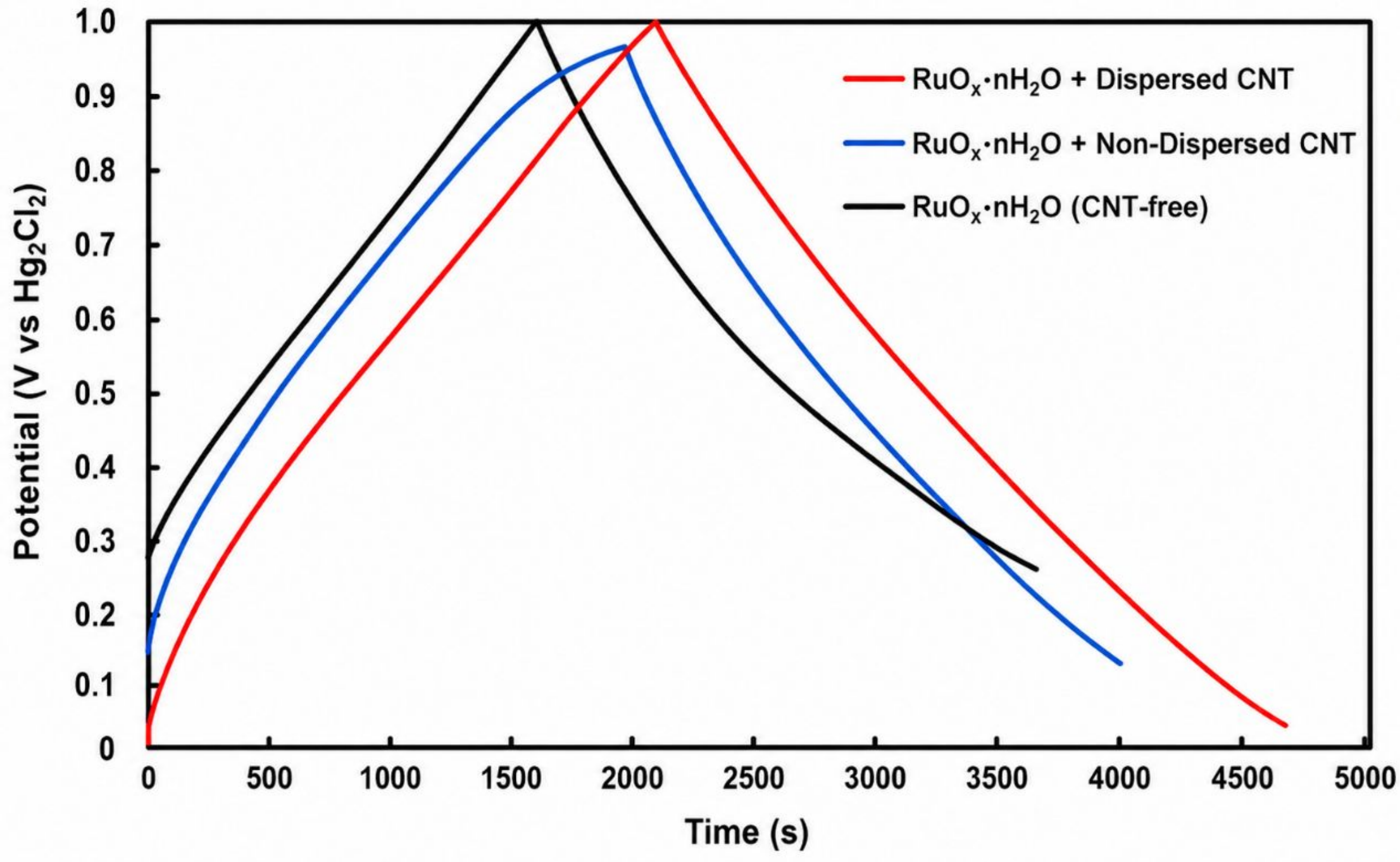

Figure S1. GCD profiles of $RuO_x \cdot nH_2O$ (CNT-free), $RuO_x \cdot nH_2O$ added non-dispersed CNTs, and $RuO_x \cdot nH_2O$ added dispersed CNTs measured at 5 mA cm^{-2}.

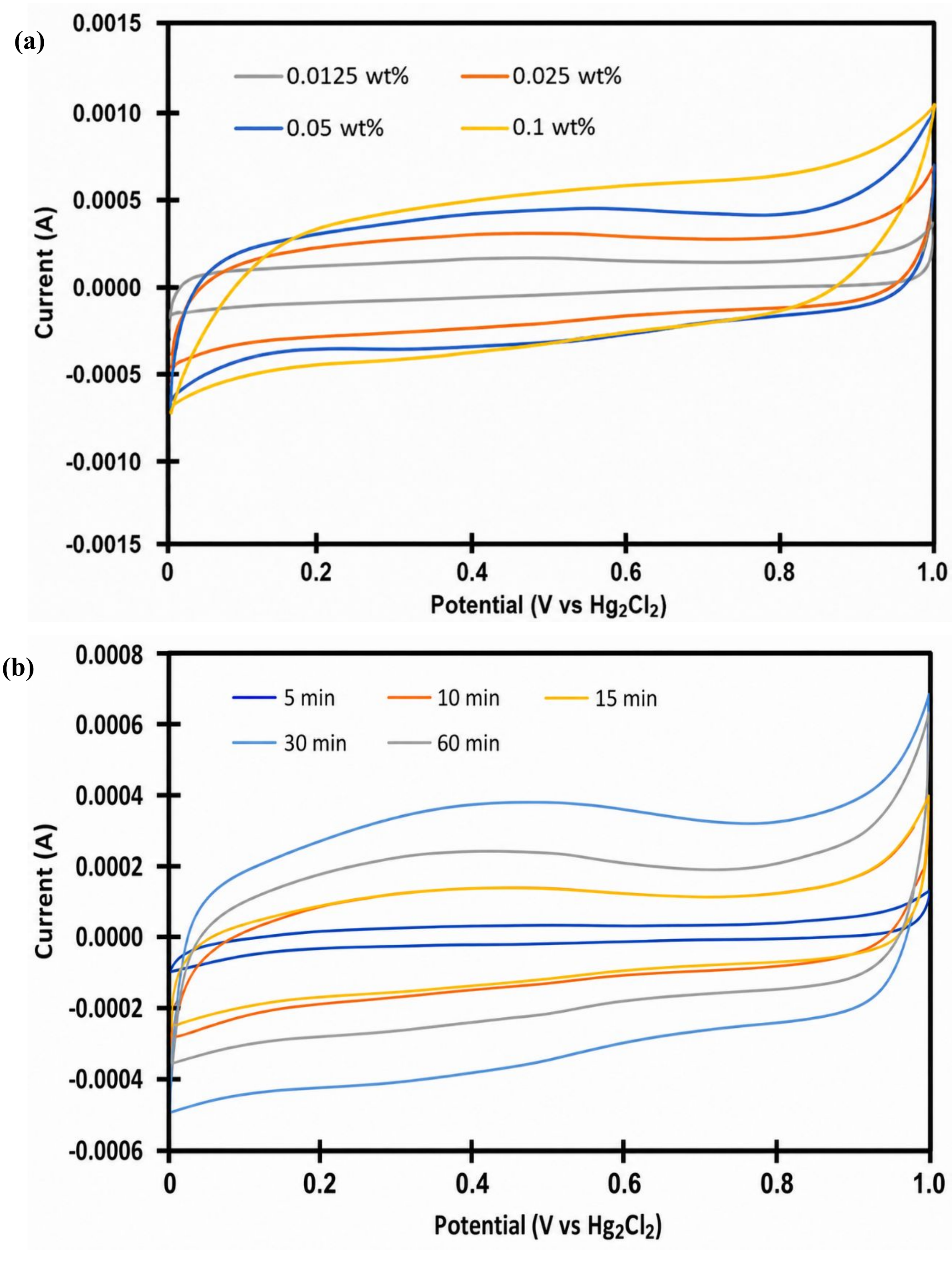

Figure S2. (a) CV curves at various CNT contents, (b) CV curves at various deposition times with 0.05 wt% dispersed CNT added.

To further evaluate the charge-storage mechanism, the relationship between current (i) and scan rate (v) was analyzed according to:

$$i = a\upsilon^{b} \quad \text{(S1)}$$

Taking the logarithm of both sides yields:

$$\log(i)=b\log(\upsilon)+\log(a) \quad \text{(S2)}$$

where *i* is the measured current and *v* is the scan rate. The b-value can be obtained from the slope of the log(i) versus log(υ) plot.

Generally, a b-value of approximately 0.5 indicates a diffusion-controlled process, whereas a value close to 1.0 corresponds to a surface-controlled capacitive process. Intermediate values ($0.5 < b < 1.0$) suggest mixed charge-storage behavior involving contributions from both mechanisms. Based on the anodic current measured at 1.0 V, a b-value of approximately 0.93 was obtained, indicating that the charge-storage process is predominantly surface-controlled. This result is consistent with the pseudocapacitive characteristics of hydrous $RuO_x{\cdot}nH_2O$ electrodes, where charge storage is primarily governed by rapid and reversible surface redox reactions.

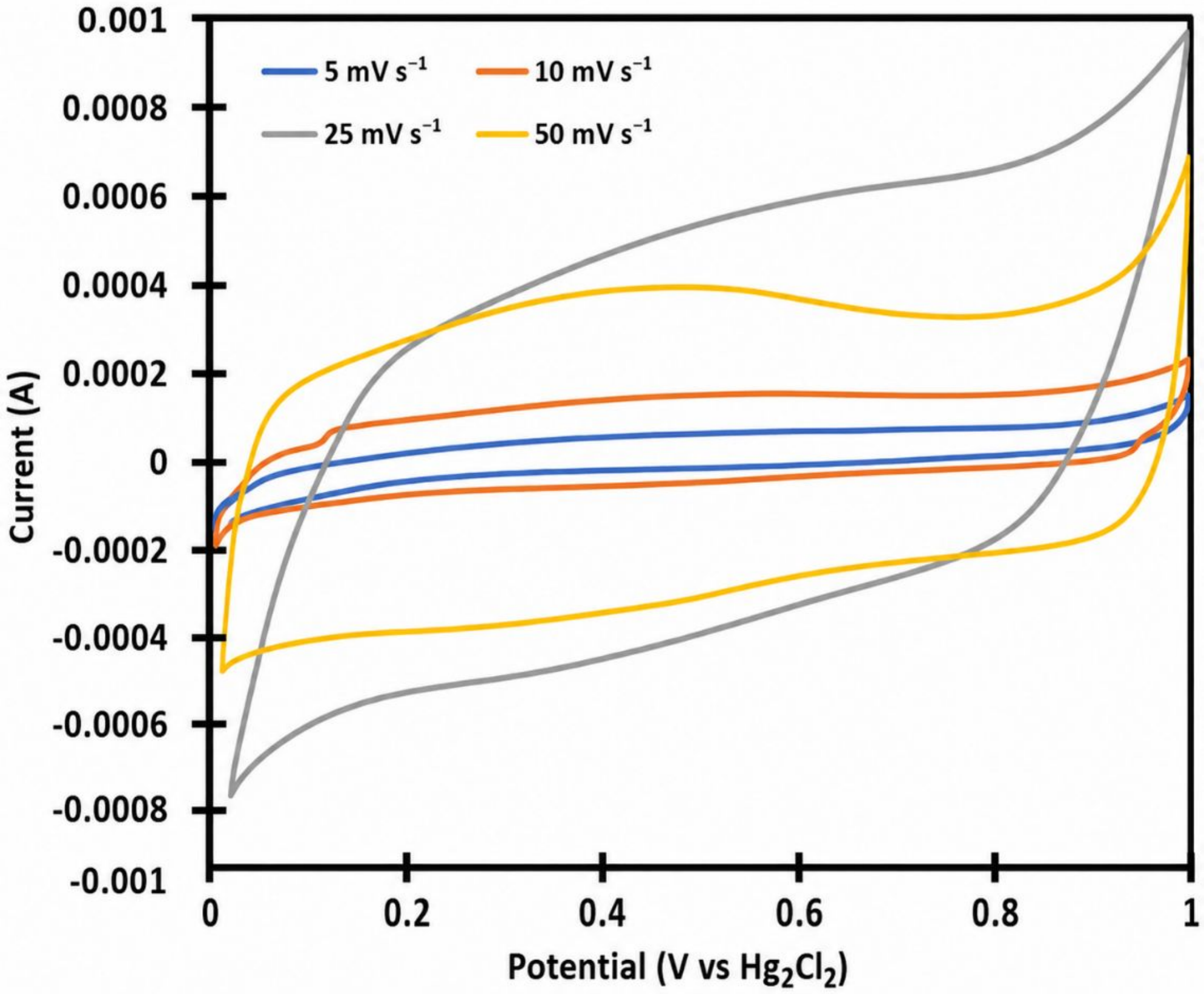

Figure S3. Determination of the b-value from CV measurements recorded at scan rates of 5, 10, 25, and 50 mV s^{-1}. The b-value was obtained from the slope of the log(i) versus log(υ) plot using the anodic current measured at 1.0 V. A b-value of approximately 0.93 indicates predominantly surface-controlled pseudocapacitive charge storage.

(a)

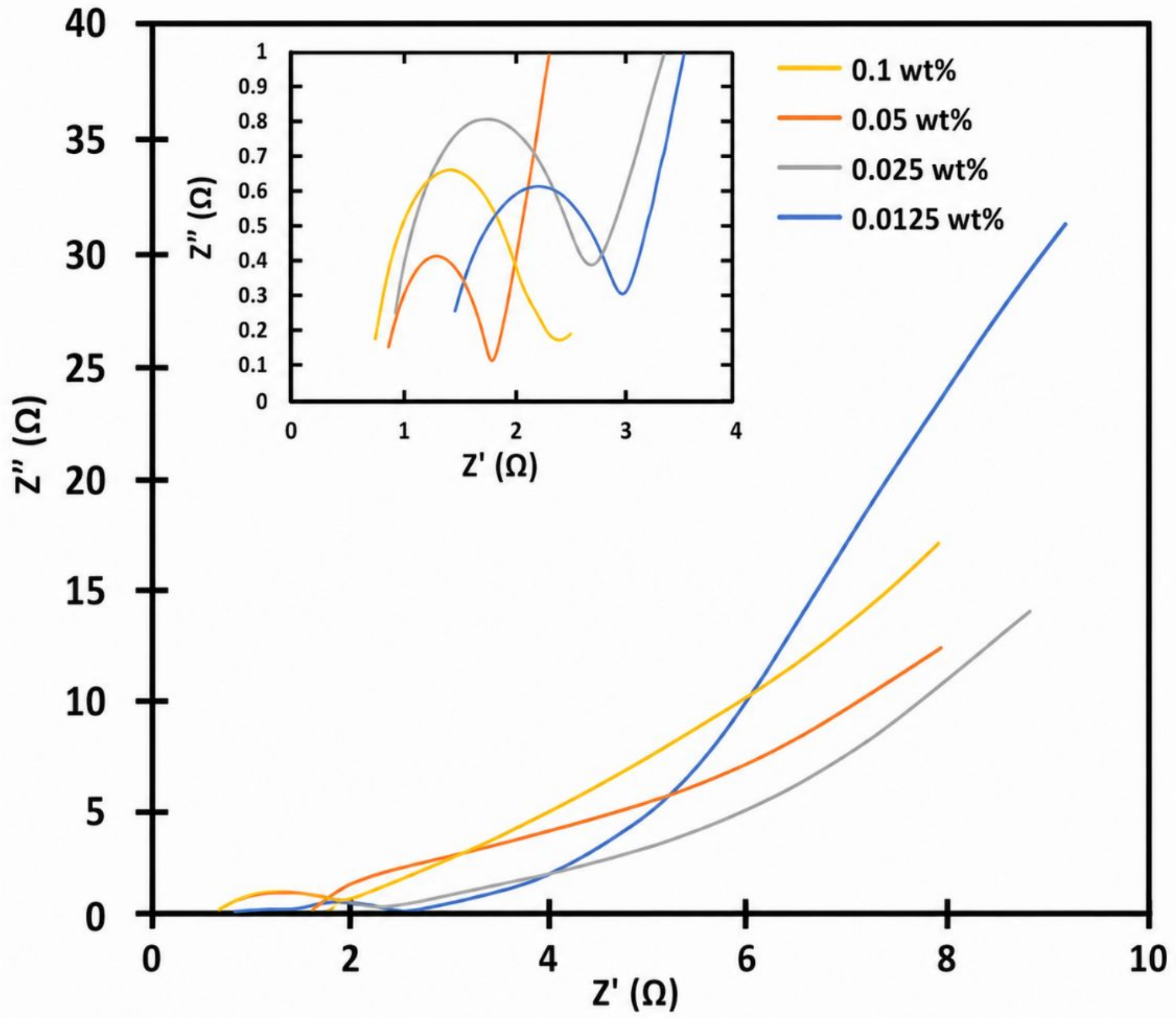

(b)

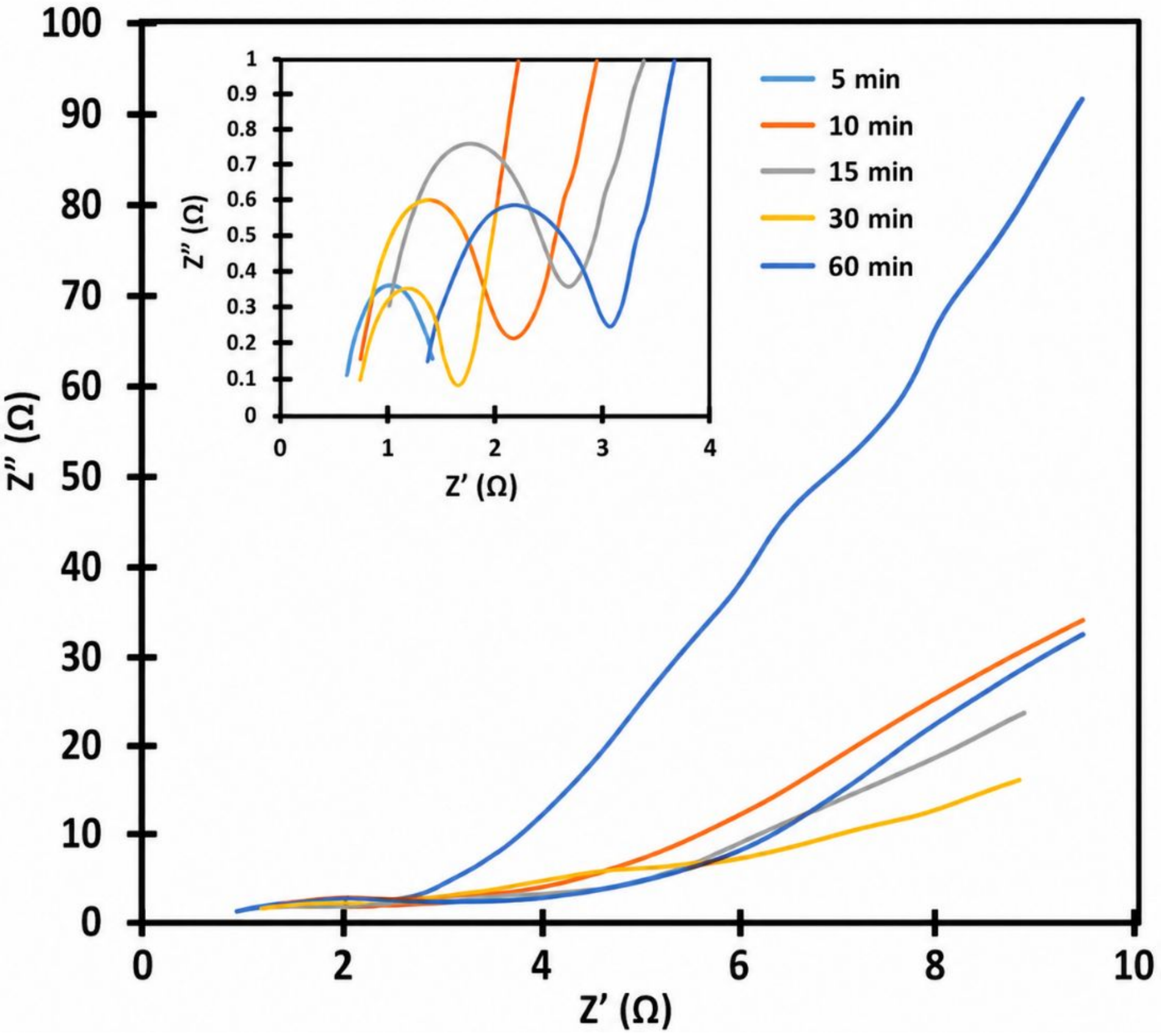

Figure S4. (a) Deposited 30 min with various contents of dispersed CNT added; (b) Added 0.05 wt% dispersed CNT with various deposited time.

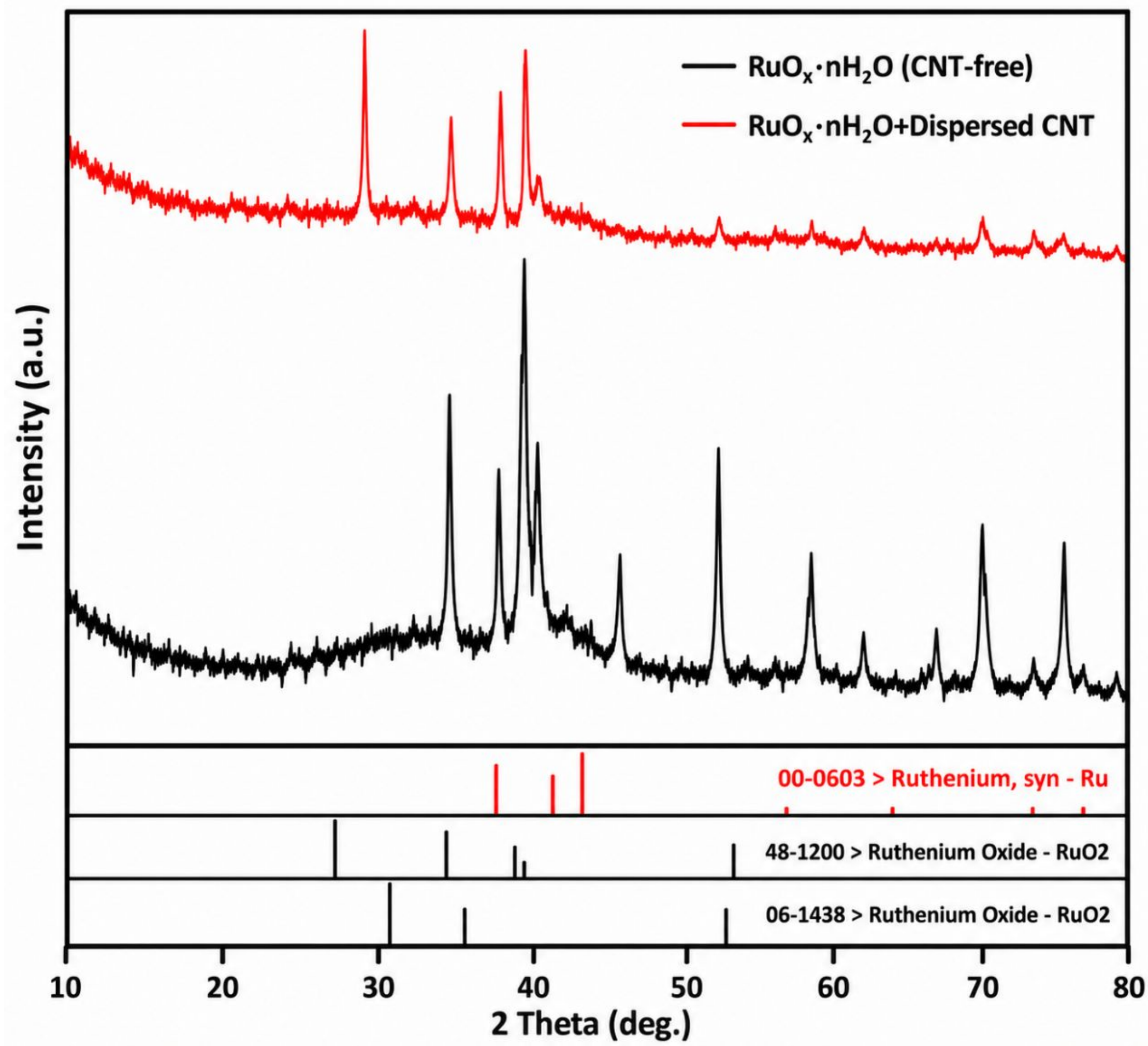

Figure S5. XRD patterns of $RuO_x{\cdot}nH_2O$ (CNT-free) electrodes and added dispersed CNTs. The incorporation of added dispersed CNTs results in reduced diffraction intensity and broader peaks, suggesting a more finely distributed and less crystalline $RuO_x{\cdot}nH_2O$ structure. No additional impurity phases are observed.

Table S1. Active material loading of $RuO_x \cdot nH_2O$ electrodes.

Electrode	Loading (mg cm^{-2})
CNT-free	0.630
Non-dispersed CNT	0.827
Dispersed CNT	1.330

Table S2. XPS fitting parameters of $RuO_x \cdot nH_2O$ electrode.

Region	Component	Binding Energy (eV)	Area (CPS)	Area Ratio	Assignment
Ru 3d	Ru $3d_5/_2$	280.94	63625.49	1.00	Ru^{4+}
Ru 3d	Ru $3d_3/_2$	285.11	52955.52	0.83	Ru^{4+}
O 1s	Ru–O	530.70	71254.67	1.00	lattice oxygen
O 1s	Ru–OH/H_2O	531.69	28982.74	0.41	hydroxyl / adsorbed water

Table S3. Apparent Rs and Rct values estimated from Nyquist plot analysis.

Specimen	Rs (Ω)	Rct (Ω)
No CNT	1.50	1.50
Non-dispersed CNT	0.76	1.48
Dispersed CNT	0.90	0.90
